# Switching Back to Normal Diet Following High-Fat Diet Feeding Reduces Cardiac Vulnerability to Ischaemia and Reperfusion Injury

**DOI:** 10.1159/000366323

**Published:** 2014-09-16

**Authors:** Ben Littlejohns, Hua Lin, Gianni D. Angelini, Andrew P. Halestrap, M. Saadeh Suleiman

**Affiliations:** ^a^Bristol Heart Institute, School of Clinical Sciences, Faculty of Medicine and Dentistry, University of Bristol, UK; ^b^School of Biochemistry, Faculty of Medical and Veterinary Sciences, University of Bristol, Bristol, UK

**Keywords:** Ischaemia/reperfusion, High-fat diet, Mice, Heart, Catalase, Mitochondrial morphology, Cardiomyocyte, Calcium transient, Twitch contraction

## Abstract

**Background:**

We have recently shown that hearts of mice fed high-fat diet exhibit increased vulnerability to ischaemia and reperfusion (I/R) in parallel to changes in catalase protein expression, mitochondrial morphology and intracellular diastolic Ca^2+^.

**Aims:**

To determine whether switching from high-fat back to normal diet alters vulnerability to I/R and to investigate cardiac cellular remodelling in relation to the mechanism(s) underlying I/R injury.

**Methods and Results:**

Male C57BL/6J mice were fed a high-fat diet for 19-22 weeks; after which a subset of mice was switched back to normal diet for 4-6 weeks. Hearts from mice switched back to normal diet were more resistant to reperfusion injury compared to hearts from mice fed only high-fat diet. This was associated with a significant reversal in catalase expression (western blotting) and recovery of size and density of mitochondria (electron microscopy). In contrast, switching back to normal diet did not alter cardiomyocyte contractility or Ca^2+^ transients compared to high-fat diet.

**Conclusion:**

This study shows for the first time that switching the diet from high-fat back to normal reduces vulnerability to I/R. This effect is associated with changes in catalase levels and mitochondrial morphology without altering cardiomyocyte contractility or Ca^2+^ transients.

## Introduction

We have recently shown that hearts from mice fed high-fat diet, in the absence of obesity associated co-morbidities, exhibit increased vulnerability to ischaemia and reperfusion (I/R) [1]. This was attributed to altered oxidative state, mitochondrial modifications and changes in Ca^2+^ handling. Interestingly, obesity-generating high-fat diet has also been shown to increase vulnerability of hearts to I/R [2,3,4]. Whether the high-fat diet increased vulnerability to I/R can be reversed by returning back to a normal diet for a relatively short period is not presently known. However, there is evidence showing that switching diet back to normal after periods of high-fat diet feeding is associated with changes back to normal conditions [5]. Such changes depend on the duration and magnitude of weight gain –factors that are often confounded because different degrees of obesity are created by varying the length of exposure to the high energy diet. For example, generating varying degrees of weight gain before switching back to normal diet can normalise metabolic abnormalities [6]. Furthermore, obesity resulting from short periods of high-fat feeding could not be fully reversed in terms of body weight and fat even after weeks of switching back to normal diet. In contrast, more recent evidence has shown that returning mice to normal diet after a long period of obesogenic high-fat diet resulted in a return of the weight to the normal level [7]. Nonetheless, several studies have demonstrated the reversibility of body weight gain when mice fed high-fat diet were switched back to normal diet [8,9]. Other key changes in the left ventricle were also reversed upon returning to normal diet including markers of inflammation, oxidised proteins, markers of apoptosis, anti-oxidants and anti-fibrotic markers [7]. High-fat diet induced changes in blood chemistry (e.g. hyperglycaemia, hyperinsulinaemia, hypertriglyceridaemia and hypercholesterolemia) are also fully reversible by returning to normal diet [9].

These studies demonstrate that many of the changes associated with obesity and high-fat diet are reversible. However, to the best of our knowledge, there are no reports showing that switching from high-fat (with or without obesity) to normal diet can reverse the increased vulnerability to I/R. Therefore, the aim of this work was to determine whether, in our recently characterised mouse model of non-obesogenic high-fat diet [1], the vulnerability to I/R can be changed by switching back to normal diet. We have also monitored myocardial cellular remodelling with emphasis on changes in catalase expression, mitochondrial morphology and Ca^2+^ handling. The reason for focusing on these endpoints was because they were the ones that correlated with increased vulnerability to I/R in the high-fat diet group [1].

## Materials and Methods

More details of materials and methods can be found in our earlier publication [1].

### Animals and Diet

Breeding, maintenance and feeding of C57BL/6J male mice as well as weight monitoring and clinical chemistry were all carried out at Charles River facilities (Charles River, Margate, UK). A summary of the feeding protocols is shown in Scheme 1. During the feeding protocols the mice were given *ad libitum* access to food and water and maintained on a 12 h light/dark cycle. Mice were fed standard murine chow diet post weaning until 6 weeks of age and they were either maintained on the standard chow (normal diet mice) or they were switched to high-fat diet (high-fat diet mice) for a further 20-22 weeks. The normal diet contained 13 % calories from fat, 22 % calories from protein and 65 % calories from carbohydrate. The high-fat diet contained 0.17 % calories from cholesterol and low sucrose content and consisted of (% calories) 45 % fat, 18 % protein and 37 % carbohydrate (Special Diets Services, UK, code: 821424). The gross energy content for high-fat and normal diets was 19.67 and 16.54 kJ· g^-1^, respectively. The detailed components of the diet can be found in [1]. A group of these mice were switched back to normal diet for 4-6 weeks after 19-20 weeks of high-fat diet (high-fat/normal diet mice). The experiments in this additional group of mice were carried out simultaneously with the two previous groups of mice which we have already published the data for [1]. The feeding duration in the high-fat/normal group was slightly different to avoid using additional groups of mice. Essentially it was a balance between keeping the feeding duration similar and having a similar overall age of the mice. As the mice were all adults, the impact of age differences is minimal.

**Figure:**
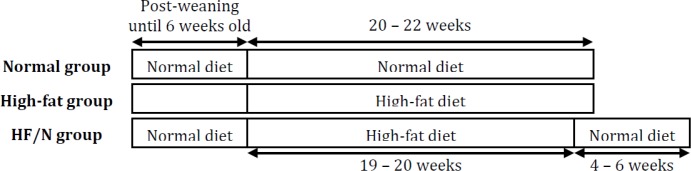

**Scheme 1.** The feeding protocols. HF/N = high-fat followed by normal diet

### Clinical chemistry measurements

Tail vein blood was taken from non-fasted mice and pooled (one pooled sample was from three animals) to measure blood chemistry components performed by Charles River (Margate, UK).

### I/R injury in isolated perfused hearts

Excised hearts were cannulated in a Langendorff mode and perfused at a constant filling pressure of 60 mmHg with Krebs-Henseleit buffer and gassed with 95 % O_2_ and 5 % CO_2_ (pH 7.4, 37°C). The Krebs-Henseleit buffer contained, in mM, 120 NaCl, 25 NaHCO_3_, 11 D-glucose anhydrous, 1.2 KH_2_PO_4_, 1.2 MgSO_4_· 7H_2_O, 4.8 KCl and 2 CaCl_2_. After a 30 min stabilisation period the hearts were rendered ischaemic for 40 min (no flow global ischaemia) followed by 120 min reperfusion. Triphenyl tetrazolium chloride (TTC) staining was used to determine the infarct size as described previously [10]. Vascular dysfunction was monitored by comparing the extent of recovery in coronary flow rate. Coronary effluent was collected at different time points and used to measure creatine kinase using a commercially available kit from Randox, UK (CK113). Total creatine kinase release was determined by calculating the area under the curve.

### Western blotting

Primary antibodies used were catalase (1:2000, Abcam) and the loading control glyceraldehyde-3-phosphate dehydrogenase (GAPDH) (1:10,000, Cell Signaling). Blots were then incubated with an appropriate horseradish peroxidase conjugated secondary antibody (1:10,000, GE Healthcare Life Sciences) and proteins were visualised using the enhanced chemiluminescence system. Protein bands were quantified by densitometry with ImageJ 1.46r software.

### Measurement of cardiac mitochondrial morphology

Mitochondrial morphology was assessed as described previously [1]. In brief, fixed cardiac tissue was processed for electron microscopy and cut into 70 nm sections with the cardiomyocytes in longitudinal plane. The mitochondria were viewed with a Tecnai 12 bioTWIN transmission electron microscope (FEI, Netherlands) and analysed using ImagePro Plus software (Media Cybernetics, USA).

### Work on isolated cardiomyocytes

Ventricular cardiomyocytes were isolated and superfused at a rate of 1.3 mL· min^-1^ with HEPES buffer solution (32-33°C), consisting of in mM, 137 NaCl, 5 KCl, 1.2 MgSO_4_· 7H_2_O, 1.2 NaH_2_PO_4_· 2H_2_O, 20 HEPES, 15 D-glucose anhydrous and 2 CaCl_2_ (pH 7.4) [1,11]. Superfused cardiomyocytes were field stimulated and contractility measured using an edge tracking device. The ratiometric dye Fura-2 was used to monitor intracellular Ca^2+^ concentrations ([Ca^2+^]_i_) and was measured using photometry (Photon Technology International, USA).

### Data analysis

Data were analysed using Prism 5 Version 5.01 software (GraphPad, USA) and presented as mean ± SEM where appropriate. Data were tested for normal distribution using Kolmogorov-Smirnov test and equal variance using F-test. One-way ANOVA with Bonferroni's post-hoc test was performed or if the data did not have equal variance and normal distribution then Kruskal-Wallis test with Dunn's post-hoc test was performed. Two-way ANOVA with Bonferroni's post-hoc test was used on data with two independent variables. All statistical tests were performed as unpaired, two-tailed and a P-value less than 0.05 was assumed to be significantly different.

## Results

This study represents an extension of our recently published work investigating the effect of high-fat diet (independent of obesity associated co-morbidities) on cardiac remodelling and vulnerability to I/R [1]. The data presented in this paper are largely focused on a group of mice fed high-fat diet (19-20 weeks) and then switched back to normal diet (4-6 weeks). For the sake of clarity, we used some of the published data from mice fed normal and high-fat diet. We have permission to use this data and this has been pointed out and acknowledged in the text.

### Characteristics and blood chemistry analysis of mice fed high-fat/normal diet

Feeding C57BL/6J male mice a non-obesogenic high-fat diet for approximately 20 weeks slightly increased the body weight and this was reversed in the high-fat/normal diet mice (Table 1). Despite the body weight returning back to the normal diet range, the epididymal fat pads were relatively lower than the high-fat diet group but still significantly heavier than the normal diet (Table 1). There was a significant reduction in blood cholesterol levels in the high-fat/normal diet compared to the high-fat diet and blood triglyceride levels in the high-fat/normal diet group were no longer different to the normal diet group (Table 1). The wet heart weight and non-fasting blood glucose were similar between the normal, high-fat and high-fat/normal diets (Table 1). The normal and high-fat diet data was taken from [1].

### The effects of I/R on hearts isolated from mice fed high-fat/normal diet

**Fig. 1:**
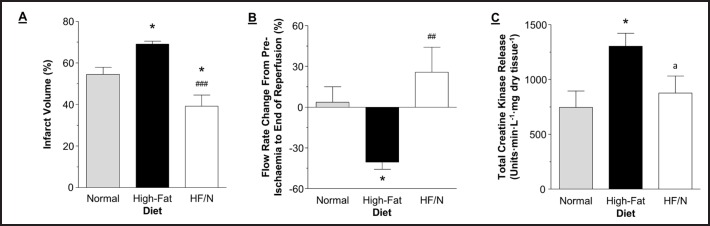
Markers of injury during I/R. A) Infarct volume quantified from the heart slices. B) Flow rate change from pre-ischaemia to the end of reperfusion. C) Total creatine kinase release collected in the coronary effluent during reperfusion. Data are presented as mean ± SEM (n = 5-7 hearts). HF/N = high-fat/normal diet. Data were analysed using one-way ANOVA with Bonferroni's post-hoc test. * = P < 0.05 vs. normal diet and ### = P < 0.001 and ## = P < 0.01 vs. high-fat diet. a is P = 0.05 vs. high-fat diet. The infarct data for normal and high-fat diets were taken from [1].

Hearts isolated from mice fed high-fat/normal diet and subjected to I/R had significantly less infarct volume compared to the high-fat diet group, P < 0.001 and lower than normal, P < 0.05 (Fig. 1 A). This was associated with significantly better preservation of coronary flow rate, P < 0.01 (Fig. 1 B) and a total release of creatine kinase which was similar to the value for normal diet (Fig. 1 C).

### Cardiac catalase protein expression in mice fed high-fat/normal diet

**Fig. 2:**
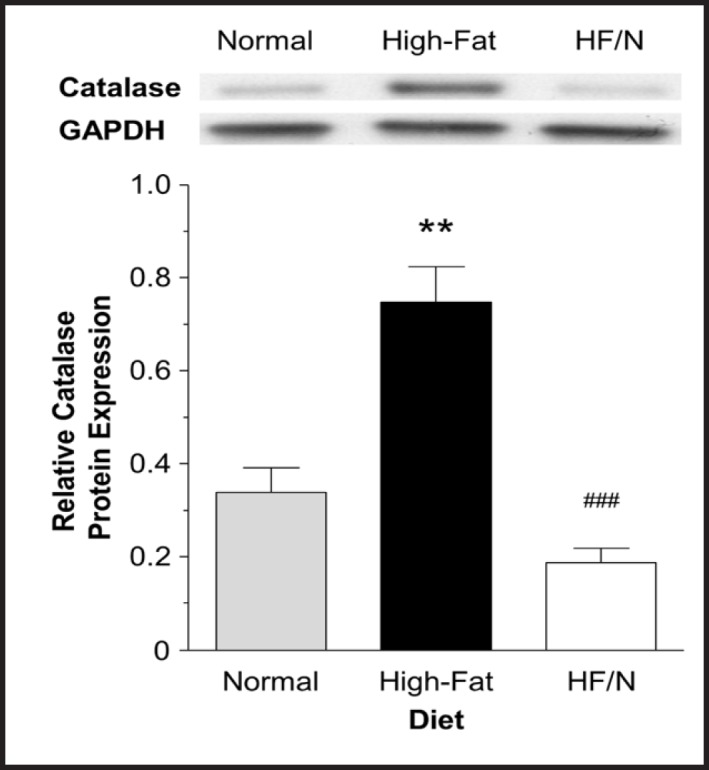
Cardiac catalase protein expression. Cardiac catalase protein expression determined using western blot. Catalase was normalised to GAPDH. Data are presented as mean ± SEM (n = 3-6 hearts). HF/N = high-fat/normal diet. Data were analysed using one-way ANOVA with Bonferroni's post-hoc test. ** = P < 0.01 vs. normal diet and ^###^ = P < 0.001 vs. high-fat diet.

In the high-fat/normal diet group the cardiac catalase protein expression was significantly lower compared to the high-fat diet group and tended to be lower than in normal diet although this was not statistically significant (Fig. 2).

### Cardiac mitochondrial morphology in mice fed high-fat/normal diet

**Fig. 3:**
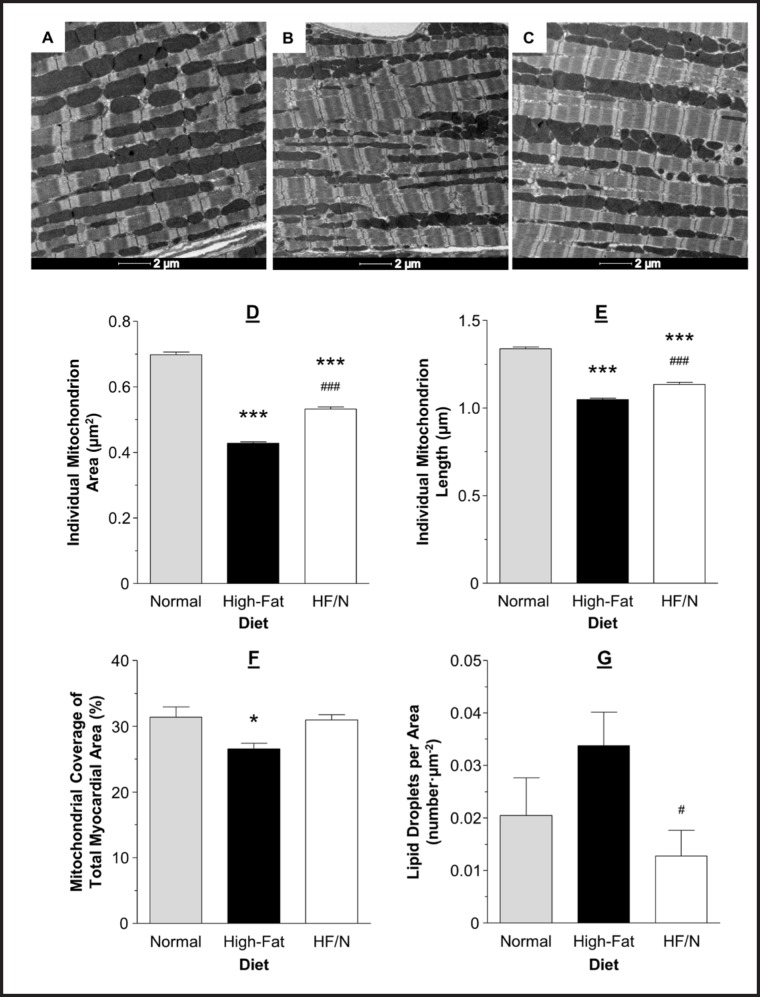
Representative electron micrographs and characteristics of mitochondrial morphology. Representative electron micrographs from hearts of mice fed normal (A), high-fat diet (B) and high-fat/normal diet (C). Individual mitochondrion area (D) and length (E), mitochondrial coverage of myofilament area (F) and number of lipid droplets per unit area (G) assessed using transmission electron micrographs. Data are presented as mean ± SEM (n = 4 hearts and ≥ 900 mitochondria per heart from ≥ 10 electron micrographs per heart). HF/N = high-fat/normal diet. Data were analysed using Kruskal-Wallis test with Dunn's post-hoc test (D-E) and one-way ANOVA with Bonferroni's post-hoc test (F-G). *** = P < 0.001 and * = P < 0.05 vs. normal diet and ^###^ = P < 0.001 and ^#^ = P < 0.05 vs. high-fat diet. The data for mitochondrial area, length and coverage for normal and high-fat diets were taken from [1].

The mitochondria in the high-fat/normal diet group were significantly larger and longer compared to the high-fat diet group, P < 0.001 (Fig. 3 B-E). This was a partial recovery as the mitochondria in the high-fat/normal diet group were still smaller and shorter compared to the normal diet, P < 0.001 (Fig. 3 A-E). The mitochondrial density (the total mitochondrial area as a percentage of total myofilament area) was increased in the high-fat/normal diet group compared to the high-fat diet group, but similar to the normal diet group (Fig. 3 F). There was a drop in the number of lipid droplets seen in electron micrographs between the high-fat diet and high-fat/normal diet groups, P < 0.05 (Fig. 3 G).

### Contractility and Ca^*2+*^ transients in cardiomyocytes isolated from mice fed high-fat/normal diet

**Fig. 4:**
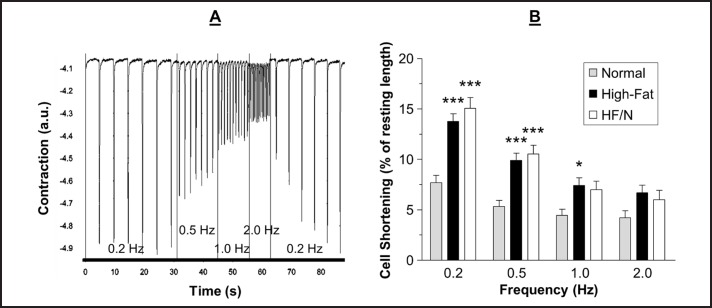
Representative contractility trace and analysis. Data were recorded using an edge tracking device to measure contractility of cardiomyocytes field stimulated at 0.2, 0.5, 1.0 and 2.0 Hz. A) Representative contractility trace of cardiomyocytes isolated from mice fed a high-fat/normal diet with the frequencies of stimulation indicated. B) The shortening of cardiomyocytes at different frequencies. Data are presented as mean ± SEM (n = 22-41 cardiomyocytes from 5-6 hearts). HF/N = high-fat/normal diet and a.u. = arbitrary units. Data were analysed using two-way ANOVA with Bonferroni post-hoc test. *** = P < 0.001 and * = P < 0.05 vs. normal diet.

A representative trace of cardiomyocyte contractility is shown in Fig. 4A. The twitch contractions of the cardiomyocytes were significantly larger in the high-fat diet group compared to the normal diet group, except at 2.0 Hz, and this difference was maintained in the high-fat/normal diet group at 0.2 and 0.5 Hz, P < 0.05 (Fig. 4 B). The time to the peak contraction and time to 90 % recovery of the resting cell length following a contraction were not different between the normal, high-fat diet and high-fat/normal diet groups, except that at 0.2 Hz the time to the contraction peak was slower in the high-fat/normal diet compared to the normal diet and high-fat diet groups (data not shown).

**Fig. 5:**
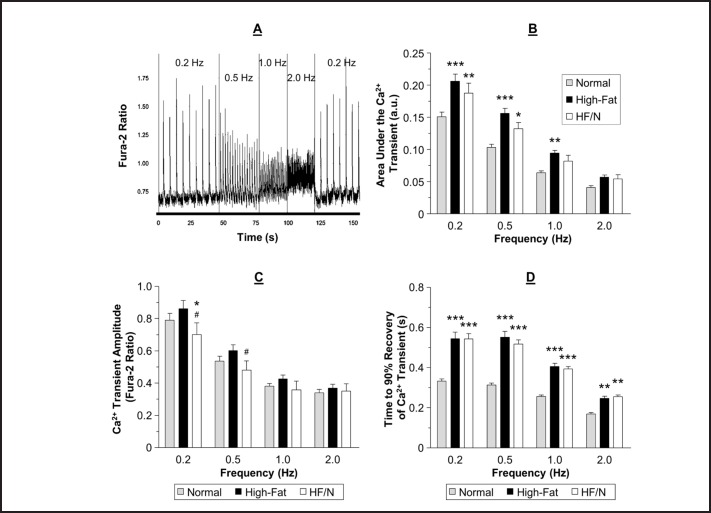
Representative Ca^2+^ transient trace and analysis. Data were recorded using Fura-2 AM fluorescence to measure Ca^2+^ transients of cardiomyocytes field stimulated at 0.2, 0.5, 1.0 and 2.0 Hz. A) Representative Ca^2+^ transient trace of cardiomyocytes isolated from mice fed a high-fat/normal diet with the frequencies of stimulation indicated. B) The area under the Ca^2+^ transient. C) The amplitude of the Ca^2+^ transient. D) Time for the Ca^2+^ transient to return to 90 % of the diastolic level. Data are presented as mean ± SEM (n = 22-32 cardiomyocytes from 3-4 hearts). HF/N = high-fat/normal diet and a.u. = arbitrary units. Data were analysed using two-way ANOVA with Bonferroni post-hoc test. *** = P < 0.001, ** = P < 0.01 and * = P < 0.05 vs. normal diet and ^#^ = P < 0.05 vs. high-fat diet.

For a representative trace of Ca^2+^ transient measurements see Fig. 5A. The area under the Ca^2+^ transient was significantly larger in the high-fat diet group compared to the normal diet group, except at 2.0 Hz, and this difference was maintained in the high-fat/normal diet group at 0.2 and 0.5 Hz, P < 0.05 (Fig. 5 B). There was no difference in the amplitude of the Ca^2+^ transient between the normal and high-fat diets. However, the amplitude was lower in the high-fat/normal diet group at 0.2 Hz compared to the both other groups and at 0.5 Hz compared to the high-fat diet group (Fig. 5 C). The normal and high-fat diets had a similar time to peak at all frequencies tested. The only frequency that the high-fat/normal diet had a slower time to peak of the Ca^2+^ transient was at 0.5 Hz (data not shown). The time to 90 % recovery of the Ca^2+^ transient was significantly longer in the high-fat diet compared to normal diet and this difference was maintained in the high-fat/normal diet group, P < 0.05 (Fig. 5 D). After feeding mice the high-fat/normal diet the diastolic [Ca^2+^]_i_ remained at the level of the high-fat diet group (data not shown) which is higher compared to normal diet (see [1]).

## Discussion

We recently used a non-obesogenic (little weight gain and no insulin resistance) mouse model of high-fat feeding (raised blood cholesterol and triglycerides) to show that isolated hearts from these mice have increased vulnerability to I/R compared to mice fed a normal diet [1]. The proposed mechanism for increased I/R injury was a combination of elevated catalase level and inability to adapt to an increase in oxidative stress, fragmentation of mitochondria, reduced mitochondrial density and elevated diastolic [Ca^2+^]_i_[1]. In this study we show for the first time that switching the diet from high-fat back to normal for a few weeks significantly reduces the vulnerability of isolated hearts to I/R. This period of normal diet was associated with reduced blood lipids, reversal of catalase protein expression and alterations in mitochondrial morphology towards that of mitochondria in normal diet mice. However, the contractile characteristics and Ca^2+^ handling measurements in cardiomyocytes remained similar to the high-fat diet.

### Switching from high-fat back to normal diet reduces vulnerability to I/R: the role of catalase and mitochondrial morphology

In this study, we found that switching the diet from high-fat to normal significantly reduces vulnerability to I/R. Reactive oxygen species (ROS) production and Ca^2+^ loading are key triggers of reperfusion injury [12]. Key in combating the increase in ROS levels are the anti-oxidant enzymes including catalase, superoxide dismutase, peroxiredoxins and glutathione peroxidase. In our earlier work, we found that high-fat diet increases the protein expression of cardiac catalase and that the level of this enzyme does not change during I/R. This was in contrast to normal diet, where this anti-oxidant enzyme markedly increased during I/R and improved the anti-oxidant capacity in the myocardium as demonstrated by relatively less increase in lipid peroxidation [1]. Therefore it was suggested that the inability of catalase to be further augmented during I/R in the high-fat diet group would reduce the capacity of the myocardium to scavenge ROS produced during I/R injury. In this study, we found that switching the diet from high-fat back to normal significantly reduced catalase protein expression back to the normal diet level (Fig. 2). This suggests that these hearts will have an improved anti-oxidant reserve and reduced susceptibility to I/R [1]. Although there are other reports showing that high-fat diet triggers an up regulation of catalase protein expression and activity [13] we are unaware of any reports showing that this can be reversed by switching back to normal diet. An interesting observation was the finding that cardiac catalase levels in the high-fat/normal diet group tended to reverse past the normal diet level. Moreover, the infarct size is actually smaller in the high-fat/normal diet compared to the normal and the recovery in flow rate also tended to be better the normal. Whether catalase is responsible for this effect is not presently known and this requires further investigation.

Altered mitochondrial morphology has been demonstrated to have an impact on vulnerability to I/R. Larger mitochondria are implicated in better protection against I/R possibly by being able to accommodate more Ca^2+^ before mitochondrial permeability transition pore opening which is a key determinant of I/R injury [12,14]. In addition, mitochondrial fragmentation was shown to be responsible for I/R injury [15]. This is consistent with our earlier study showing that high-fat diet was associated with smaller and shorter mitochondria (fragmented mitochondria) and increased I/R injury [1]. Furthermore, we found high-fat diet to be associated with less mitochondrial coverage of myofilament area. Thus it was concluded that high-fat diet altering of mitochondrial morphology/density predisposes the heart to reperfusion injury. In this study, we found that switching the diet back to normal, significantly reversed these changes (Fig. 3) and therefore might be responsible for improved cardioprotection. These changes were only a partial recovery as the mitochondria were still shorter and smaller compared to the normal diet; however, despite the mitochondria being smaller and shorter the density had returned to the normal diet level.

The changes in catalase and mitochondrial morphology are reversed in the high-fat/normal diet group compared to high-fat diet and these are likely to be responsible for decreased I/R injury. However, the possibility that other cellular changes also contribute to this cannot be excluded.

### Cardiomyocyte Ca^*2+*^ transients and contractility do not change in response to switching from high-fat back to normal diet

In our earlier work we found that high-fat diet was associated with a raised cytosolic Ca^2+^ content in cardiomyocytes compared to the normal diet group and this is known to be trigger for increased I/R injury [12]. Surprisingly, switching the diet from high-fat back to normal was not associated with changes in cytosolic Ca^2+^ or in any of the characteristics of the Ca^2+^ transients suggesting that our original proposal that the high-fat diet increased vulnerability to I/R could be due to Ca^2+^ regulation may not be correct.

In this study, we report the characteristics of cardiomyocyte twitch contractions from normal, high-fat and high-fat/normal diet groups. In parallel to our finding for Ca^2+^ transients, we found that cardiomyocyte contractility changes in high-fat diet and that the changes are not reversed, on the whole, upon switching back to normal diet (Fig. 4). The cardiomyocyte twitch contractions were larger in high-fat diet compared to normal diet and this was maintained in the high-fat/normal diet group. At the higher frequencies studied the difference between the groups became smaller; a likely explanation for this could be reduced sensitivity of the detection system resulting from having smaller amplitude at higher rates. Increased amplitude of cardiomyocyte contractility has already been reported in mice fed a high-fat high-sucrose diet which induced obesity and diabetes. These mice demonstrated an increased peak shortening in cardiomyocytes isolated from mice at 5 and 7 months of high-fat feeding [16]. Heart failure induced in rats by coronary artery ligation and then fed a high saturated fat diet also had improved contractile function *in vivo* compared to the normal diet; however the saturated fat diet had no effect on contractility in the absence of heart failure [17]. The novel finding here is that by switching back to normal diet the increased amplitude of twitch contractions is not reversed.

Despite the larger twitch contractions there was no increase in the Ca^2+^ transient amplitude. However, there was a larger area under the Ca^2+^ transient which is likely to lead to the larger twitch contractions in the cardiomyocytes isolated from high-fat fed mice compared to normal diet fed mice. Other possible mechanisms for the increased twitch contractions are altered sensitivity of the myofilaments to Ca^2+^ and changes in the viscoelastic properties of the cardiomyocyte in response to high-fat diet. The greater area under the Ca^2+^ transient can be attributed to the slower reuptake of Ca^2+^ following a Ca^2+^ transient. As we have already reported, mice fed a high-fat diet have reduced cardiac phospholamban phosphorylation at site Ser16 [1]. In the high-fat/normal diet group the phospholamban phosphorylation was similar to the high-fat diet group (unpublished data). In rodents, the majority of the Ca^2+^ released from the sarcoplasmic reticulum during a Ca^2+^ transient is taken back up into the sarcoplasmic reticulum via SERCA [18]. The remaining Ca^2+^ is extruded from the cell by the Na^+^/Ca^2+^ exchanger, the Ca^2+^ ATPase or taken up into the mitochondria. The rate of the Ca^2+^ transient recovery therefore largely depends on SERCA and its activity. Phospholamban is a regulator of SERCA activity and therefore the slower Ca^2+^ transient in the high-fat diet group is likely to be due to the reduced phospholamban phosphorylation.

However, one thing to note is that the increase in amplitude of contraction seen in the high-fat diet group compared to normal diet group was not observed in echocardiographic measurements as there was no change in ejection fraction or fractional shortening (see [1]). The stimulation rate used in the isolated cardiomyocytes was far from the physiological rate seen *in vivo*; when the stimulation frequency reached 2.0 Hz there was no longer a significantly increased twitch contraction, therefore it is possible that the changes observed in the cardiomyocytes do not represent what occurs *in vivo*. It is not known what the physiological significance of the increased twitch contractions at low frequencies is. It is also not known if the Ca^2+^ and contractility changes could be reversed if the mice were maintained on the normal diet for a longer period following the high-fat diet. Nonetheless, as the vulnerability to I/R was reversed in the high-fat/normal diet group but the Ca^2+^ and contractility changes where not then this suggests they are not contributing to the I/R vulnerability in the high-fat diet group.

The mice in the high-fat/normal diet group were approximately three weeks older compared to the normal and high-fat diet groups (approx. 30 vs 27 weeks). This is only a small age difference and all groups are considered as adult mice. It is not known whether these age differences between the mice in this model had an impact on vulnerability to I/R; however, ageing has been shown to augment (not attenuate) the damaging effects of I/R [19,20].

## Conclusions

This study shows for the first time that switching the diet from high-fat back to normal reduces vulnerability to I/R. This effect is associated with changes in catalase levels and mitochondrial morphology without altering cardiomyocyte contractility or Ca^2+^ transients. Therefore, the increased vulnerability to I/R after feeding mice high-fat diet is likely to be related to the elevated catalase expression and mitochondrial fragmentation.

## Disclosure Statement

The authors declare that there are no conflicts of interest.

## Figures and Tables

**Table 1:** Characteristics of the mice and blood chemistry analysis. Data are presented as mean ± SEM. For blood for chemistry analysis each n was a pooled sample from three mice. Data were analysed using the appropriate statistical test. * = P < 0.05 vs. normal diet and # = P < 0.05 vs. high-fat diet

Measurement	Normal diet	High-fat diet	High-fat/normal diet
Body weight (g)	31.2 ± 0.2 (n = 158)	32.2 ± 0.3* (n = 153)	31.5 ± 0.4 (n = 32)
Epididymal fat pad weight (g)	0.53 ± 0.02 (n = 55)	1.19 ± 0.11* (n = 34)	0.79 ± 0.08* (n = 11)
Wet heart weight (mg)	219 ± 5 (n = 55)	215 ± 6 (n = 34)	216 ± 7 (n = 11)
Cholesterol (mM)	3.25 ± 0.20 (n = 4)	5.06 ± 0.52* (n = 4)	3.43 ± 0.29^#^ (n = 4)
Triglycerides (mg-dL^−1^)	120 ± 15 (n = 4)	205 ± 26* (n = 4)	156 ± 16 (n = 4)
Non-fasting glucose (mM)	9.26 ± 0.64 (n = 4)	7.54 ± 0.72 (n = 4)	8.90 ± 0.40 (n = 4)
